# Supplementary material for: Physiological effects of filtering facepiece respirators based on age and exercise intensity
Source: PLoS One. 2024 Aug 29;19(8):e0309403. doi: 10.1371/journal.pone.0309403 (PMC11361601; doi:10.1371/journal.pone.0309403)
Supplement: S1 Table — (DOCX) [file pone.0309403.s001.docx]

| **S1 Table.** **The modified Bruce protocol (Exercise sessions).** | | | |
| --- | --- | --- | --- |
| Duration (min) | Speed (km/hr) | Incline (%) |  |
| 3 | 2.74 | 0 |  |
| 3 | 2.74 | 5 |  |
| 3 | 2.74 | 10 |  |
| 3 | 4.02 | 12 |  |
| 3 | 5.47 | 14 |  |
| 3 | 6.76 | 16 |  |
| *The modified Bruce protocol: One of the treadmill stress test protocols to evaluate the exercise ability of a subject while gradually increasing the exercise intensity (speed, incline). | | | |
