# Supplementary material for: Physiological effects of filtering facepiece respirators based on age and exercise intensity
Source: PLoS One. 2024 Aug 29;19(8):e0309403. doi: 10.1371/journal.pone.0309403 (PMC11361601; doi:10.1371/journal.pone.0309403)
Supplement: S2 Table — (DOCX) [file pone.0309403.s002.docx]

| **S2 Table.** **Comparison of physiological parameters at various exercies intensities in children group.** | | | | | |
| --- | --- | --- | --- | --- | --- |
| Children group |  |  | |  |  |
|  | Control  (N = 10) | Cup  (N = 10) | FF  (N = 10) | | *p* value |
| Rest |  |  |  | |  |
| Rf (breaths/min) | 18.8 ± 2.8 | 18.8 ± 2.4 | 18.6 ± 2.2 | | 0.98 |
| VE (L/min) | 11.2 ± 2.5 | 11.7 ± 2.7 | 10.4 ± 2.7 | | 0.51 |
| VCO_2_ (mL/min) | 304.0 ± 73.3 | 306.4 ± 78.0 | 291.6 ± 67.7 | | 0.89 |
| VO_2_/KG (mL/min/kg) | 5.9 ± 1.2 | 6.3 ± 0.9 | 6.2 ± 1.1 | | 0.80 |
| METs | 1.7 ± 0.4 | 1.8 ± 0.3 | 1.8 ± 0.3 | | 0.80 |
| HR (beats/min) | 86.6 ± 7.1 | 88.9 ± 9.5 | 87.1 ± 10.4 | | 0.85 |
| SpO_2_ (%) | 96.6 ± 0.8 | 97.5 ± 0.7 | 97.1 ± 1.4 | | 0.15 |
| Low intensity |  |  |  | |  |
| Rf (breaths/min) | 29.1 ± 3.3 | 28.8 ± 3.1 | 29.2 ± 2.9 | | 0.94 |
| VE (L/min) | 19.1 ± 3.2 | 20.7 ± 5.0 | 20.2 ± 5.2 | | 0.73 |
| VCO_2_ (mL/min) | 546.2 ± 96.7 | 574.2 ± 155.2 | 591.7 ± 129.2 | | 0.73 |
| VO_2_/KG (mL/min/kg) | 12.8 ± 2.3 | 13.0 ± 1.7 | 13.8 ± 1.7 | | 0.49 |
| METs | 3.7 ± 0.6 | 3.7 ± 0.5 | 3.9 ± 0.5 | | 0.49 |
| HR (beats/min) | 110.4 ± 6.1 | 109.5 ± 9.0 | 111.8 ± 8.0 | | 0.82 |
| SpO_2_ (%) | 96.6 ± 1.4 | 96.6 ± 1.0 | 95.9 ± 1.7 | | 0.42 |
| Moderate intensity |  |  |  | |  |
| Rf (breaths/min) | 34.3 ± 3.7 | 32.9 ± 2.3 | 34.4 ± 3.6 | | 0.50 |
| VE (L/min) | 28.2 ± 5.5 | 29.1 ± 6.5 | 29.5 ± 8.7 | | 0.92 |
| VCO_2_ (mL/min) | 839.5 ± 159.0 | 874.2 ± 224.3 | 922.5 ± 227.7 | | 0.67 |
| VO_2_/KG (mL/min/kg) | 18.3 ± 3.3 | 18.9 ± 2.9 | 20.0 ± 2.5 | | 0.43 |
| METs | 5.2 ± 1.0 | 5.4 ± 0.8 | 5.7 ± 0.7 | | 0.43 |
| HR (beats/min) | 126.3 ± 7.4 | 125.3 ± 11.5 | 129.7 ± 11.8 | | 0.61 |
| SpO_2_ (%) | 96.0 ± 1.2 | 95.2 ± 1.7 | 94.7 ± 2.0 | | 0.23 |
| High intensity |  |  |  | |  |
| Rf (breaths/min) | 42.4 ± 4.7 | 38.8 ± 4.9 | 41.7 ± 6.4 | | 0.30 |
| VE (L/min) | 46.0 ± 8.4 | 42.8 ± 8.8 | 45.9 ± 13.0 | | 0.73 |
| VCO_2_ (mL/min) | 1420.2 ± 275.8 | 1402.2 ± 370.0 | 1545.5 ± 386.5 | | 0.61 |
| VO_2_/KG (mL/min/kg) | 27.5 ± 5.1 | 27.6 ± 4.1 | 29.6 ± 3.2 | | 0.44 |
| METs | 7.9 ± 1.5 | 7.9 ± 1.2 | 8.5 ± 0.9 | | 0.44 |
| HR (beats/min) | 158.5 ± 12.2 | 149.5 ± 19.4 | 159.1 ± 11.5 | | 0.29 |
| SpO_2_ (%) | 94.4 ± 2.1 | 94.7 ± 2.2 | 93.7 ± 2.1 | | 0.59 |
| Recovery |  |  |  | |  |
| Rf (breaths/min) | 38.9 ± 3.9 | 36.3 ± 3.4 | 37.5 ± 3.4 | | 0.28 |
| VE (L/min) | 40.5 ± 8.6 | 37.7 ± 9.2 | 38.4 ± 11.9 | | 0.81 |
| VCO_2_ (mL/min) | 1191.9 ± 241.2 | 1194.0 ± 379.5 | 1245.2 ± 396.5 | | 0.93 |
| VO_2_/KG (mL/min/kg) | 20.0 ± 3.4 | 20.6 ± 3.4 | 20.9 ± 1.8 | | 0.76 |
| METs | 5.7 ± 1.0 | 5.9 ± 1.0 | 6.0 ± 0.5 | | 0.77 |
| HR (beats/min) | 148.0 ± 12.0 | 139.1 ± 18.0 | 146.4 ± 13.7 | | 0.37 |
| SpO_2_ (%) | 96.6 ± 1.8 | 96.6 ± 1.1 | 96.3 ± 1.6 | | 0.90 |
| The values were shown in mean ± standard deviation. Significance level was set at p <0.05. Significant results are indicated in bold.  Rf: Respiratory frequency; VE: Minute Ventilation; VCO_2_: Volume of Carbon dioxide consumed by the body per minute; VO_2_: Volume of Oxygen consumed by the body per minute; METs: Metabolic equivalent; HR: Heart Rate; SpO_2_: percutaneous oxygen saturation. | | | | | |
