# Supplementary material for: Physiological effects of filtering facepiece respirators based on age and exercise intensity
Source: PLoS One. 2024 Aug 29;19(8):e0309403. doi: 10.1371/journal.pone.0309403 (PMC11361601; doi:10.1371/journal.pone.0309403)
Supplement: S3 Table — (DOCX) [file pone.0309403.s003.docx]

| **S3 Table. Comparison of physiological parameters at various exercise intensities in young adults group.** | | | | | | |
| --- | --- | --- | --- | --- | --- | --- |
| Young adults group |  |  |  |  |  | |
|  | Control  (N = 10) | Cup  (N = 10) | FF^a^  (N = 10) | Valve^b^  (N = 6) | | *p*-value |
| Rest |  |  |  |  | |  |
| Rf (breaths/min) | 17.1 ± 3.6 | 16.9 ± 3.3 | 15.8 ± 3.7 | 15.5 ± 3.4 | | 0.72 |
| VE (L/min) | 9.8 ± 1.6 | 10.7 ± 2.8 | 10.0 ± 1.9 | 10.3 ± 1.5 | | 0.79 |
| VCO_2_ (mL/min) | 254.1 ± 59.6 | 256.9 ± 89.7 | 283.1 ± 68.9 | 249.8 ± 53.3 | | 0.74 |
| VO_2_/KG (mL/min/kg) | 4.9 ± 0.8 | 4.9 ± 0.7 | 5.3 ± 0.5 | 4.7 ± 0.9 | | 0.29 |
| METs | 1.4 ± 0.2 | 1.4 ± 0.2 | 1.5 ± 0.2 | 1.3 ± 0.3 | | 0.30 |
| HR (beats/min) | 74.8 ± 6.5 | 77.6 ± 9.0 | 76.2 ± 8.5 | 78.3 ± 5.9 | | 0.81 |
| SpO_2_ (%) | 96.6 ± 1.7 | 97.8 ± 1.1 | 96.4 ± 1.5 | 97 ± 1.1 | | 0.20 |
| Low intensity |  |  |  |  | |  |
| Rf (breaths/min) | 24.7 ± 3.7 | 21.7 ± 4.3 | 22.8 ± 3.5 | 22.1 ± 4.4 | | 0.39 |
| VE (L/min) | 20.1 ± 3.8 | 20.0 ± 4.0 | 19.8 ± 3.6 | 19.6 ± 2.8 | | 0.99 |
| VCO_2_ (mL/min) | 588.0 ± 150.0 | 588.6 ± 155.4 | 642.9 ± 150.8 | 561.9 ± 112.2 | | 0.71 |
| VO_2_/KG (mL/min/kg) | 12.1 ± 1.1 | 11.9 ± 1.2 | 12.9 ± 1.1 | 11.3 ± 1.7 | | 0.10 |
| METs | 3.5 ± 0.3 | 3.4 ± 0.4 | 3.7 ± 0.3 | 3.2 ± 0.5 | | 0.10 |
| HR (beats/min) | 92.6 ± 6.5 | 96.2 ± 9.4 | 92.3 ± 7.2 | 96.3 ± 5.3 | | 0.54 |
| SpO_2_ (%) | 96.6 ± 1.7 | 95.8 ± 1.1 | 95.9 ± 1.5 | 96.7 ± 1.1 | | 0.45 |
| Moderate intensity |  |  |  |  | |  |
| Rf (breaths/min) | 27.1 ± 4.8 | 24.4 ± 5.4 | 25.0 ± 4.7 | 25.7 ± 4.3 | | 0.62 |
| VE (L/min) | 33.6 ± 8.4 | 31.8 ± 8.3 | 31.4 ± 9.0 | 31.1 ± 6.0 | | 0.92 |
| VCO_2_ (mL/min) | 1092.3±351.1 | 1074.2±331.3 | 1183.5±383.6 | 1027.0±248.8 | | 0.82 |
| VO_2_/KG (mL/min/kg) | 20.8 ± 3.0 | 20.3 ± 3.6 | 21.8 ± 3.2 | 19.3 ± 4.0 | | 0.54 |
| METs | 5.9 ± 0.9 | 5.8 ± 1.0 | 6.2 ± 0,9 | 5.5 ± 1.2 | | 0.54 |
| HR (beats/min) | 116.3 ± 6.3 | 119.0 ± 8.8 | 115.7 ± 10.3 | 120.2 ± 12.2 | | 0.74 |
| SpO_2_ (%) | 93.9 ± 1.5 | 94.3 ± 2.1 | 94.5 ± 2.3 | 94 ± 1.7 | | 0.90 |
| High intensity |  |  |  |  | |  |
| Rf (breaths/min) | 32.5 ± 5.8 | 31.8 ± 6.3 | 30.5 ± 5.6 | 30.4 ± 5.5 | | 0.85 |
| VE (L/min) | 53.7 ± 14.2 | 51.6 ± 14.5 | 50.8 ± 16.1 | 47.5 ± 9.7 | | 0.86 |
| VCO_2_ (mL/min) | 1845.1± 558.8 | 1828.1±535.5 | 2018.4±603.5 | 1720.0±424.3 | | 0.74 |
| VO_2_/KG (mL/min/kg) | 30.5 ± 4.7 | 29.8 ± 4.9 | 32.5 ± 4.5 | 28.5 ± 6.2 | | 0.43 |
| METs | 8.7 ± 1.4 | 8.5 ± 1.4 | 9.3 ± 1.3 | 8.2 ± 1.8 | | 0.43 |
| HR (beats/min) | 147.9 ± 7.4 | 150.1 ± 9.2 | 147.4 ±9.2 | 151.2 ± 12.4 | | 0.83 |
| SpO_2_ (%) | 94 ± 2.0 | 92.4 ± 1.9 | 93.5 ± 1.3 | 93.5 ± 2.2 | | 0.34 |
| Recovery |  |  |  |  | |  |
| Rf (breaths/min) | 32.4 ± 3.8 | 31.7 ± 5.8 | 30.5 ± 5.1 | 29.0 ± 4.0 | | 0.56 |
| VE (L/min) | 48.4 ± 9.8 | 45.2 ± 8.6 | 45.3 ± 10.4 | 41.6 ± 4.8 | | 0.54 |
| VCO_2_ (mL/min) | 1512.5 ± 345.6 | 1496.7 ± 300.9 | 1665.9 ± 343.7 | 1394.0 ± 149.0 | | 0.37 |
| VO_2_/KG (mL/min/kg) | 20.5 ± 2.0 | 20.3 ± 2.5 | 22.5 ± 1.9 | 19.0 ± 2.9 | | **< 0.05** ^b<a^ |
| METs | 5.9 ± 0.6 | 5.8 ± 0.7 | 6.4 ± 0.5 | 5.4 ± 0.8 | | **< 0.05** ^b<a^ |
| HR (beats/min) | 137.0 ± 9.0 | 141.0 ± 12.1 | 137.7 ± 12.3 | 139.6 ± 7.4 | | 0.85 |
| SpO_2_ (%) | 96 ± 1.8 | 95.5 ± 2.0 | 96.2 ± 1.3 | 96 ± 1.6 | | 0.82 |
| The values are shown in mean ± standard deviation. Significance level was set at p <0.05. Significant results are indicated in bold.  Rf: Respiratory frequency; VE: Minute Ventilation; VCO_2_: Volume of Carbon dioxide consumed by the body per minute; VO_2_: Volume of Oxygen consumed by the body per minute; METs: Metabolic equivalent; HR: Heart Rate; SpO_2_: percutaneous oxygen saturation. | | | | | | |
