# Supplementary material for: Physiological effects of filtering facepiece respirators based on age and exercise intensity
Source: PLoS One. 2024 Aug 29;19(8):e0309403. doi: 10.1371/journal.pone.0309403 (PMC11361601; doi:10.1371/journal.pone.0309403)
Supplement: S4 Table — (DOCX) [file pone.0309403.s004.docx]

| **S4 Table.** **Comparison of physiological parameters at various exercise intensities in older adults group.** | | | | | |
| --- | --- | --- | --- | --- | --- |
| Older adults group |  |  |  |  |  |
|  | Control  (N = 8) | Cup  (N = 8) | FF  (N = 8) | Valve  (N = 4) | *p*-value |
| Rest |  |  |  |  |  |
| Rf (breaths/min) | 17.1 ± 2.5 | 17.2 ± 1.9 | 16.2 ± 2.4 | 17.3 ± 2.6 | 0.78 |
| VE (L/min) | 9.6 ± 2 | 11.2 ± 2.8 | 9.8 ± 2.1 | 10.7 ± 1.8 | 0.50 |
| VCO_2_ (mL/min) | 244.1 ± 41.1 | 252.3 ± 66.8 | 242.4 ± 56.9 | 220 ± 28.6 | 0.80 |
| VO_2_/KG (mL/min/kg) | 4.4 ± 0.3 | 4.5 ± 0.6 | 4.5 ± 0.8 | 4.3 ± 0.2 | 0.90 |
| METs | 1.2 ± 0.1 | 1.3 ± 0.2 | 1.3 ± 0.2 | 1.2 ± 0.0 | 0.90 |
| HR (beats/min) | 63.1 ± 4.8 | 66.2 ± 8.4 | 68.8 ± 7.4 | 62 ± 8.0 | 0.33 |
| SpO_2_ (%) | 96.8 ± 0.9 | 96.4 ± 0.7 | 95.8 ± 0.9 | 96.8 ± 1.0 | 0.12 |
| Low intensity |  |  |  |  |  |
| Rf (breaths/min) | 23.5 ± 2.0 | 21.8 ± 2.3 | 21.3 ± 2.6 | 22.5 ± 3.2 | 0.33 |
| VE (L/min) | 21.6 ± 5.5 | 21.6 ± 6.3 | 20.3 ± 4.3 | 21.1 ± 5.3 | 0.96 |
| VCO_2_ (mL/min) | 622.5 ± 135.8 | 609.8 ± 182.2 | 611.6 ± 120.3 | 580 ± 132.3 | 0.97 |
| VO_2_/KG (mL/min/kg) | 11.5 ± 0.8 | 11.1 ± 1.9 | 11.6 ± 1.4 | 11.3 ± 0.8 | 0.91 |
| METs | 3.3 ± 0.2 | 3.2 ± 0.5 | 3.3 ± 0.4 | 3.2 ± 0.2 | 0.91 |
| HR (beats/min) | 80.9 ± 6.2 | 83.2 ±7.0 | 86.8 ± 8.2 | 78.7 ± 5.8 | 0.24 |
| SpO_2_ (%) | 96.6 ± 0.5 | 95.9 ± 1.4 | 95.9 ± 1.0 | 96.5 ± 1.3 | 0.39 |
| Moderate intensity |  |  |  |  |  |
| Rf (breaths/min) | 26.9 ± 3.2 | 24.3 ± 2.5 | 24.3 ± 3.0 | 26 ± 4.9 | 0.32 |
| VE (L/min) | 32.8 ± 6.7 | 30.9 ± 8.7 | 30.5 ± 7.0 | 32.6 ± 7.8 | 0.91 |
| VCO_2_ (mL/min) | 1025.1 ± 176.6 | 979.1 ± 255.4 | 1013.7 ± 193.6 | 1020.1 ± 210.1 | 0.97 |
| VO_2_/KG (mL/min/kg) | 18.3 ± 21 | 17.5 ± 3.3 | 18.5 ± 3.1 | 19.2 ± 0.9 | 0.76 |
| METs | 5.2 ± 0.6 | 5 ± 0.9 | 5.3 ± 0.9 | 5.5 ± 0.3 | 0.75 |
| HR (beats/min) | 98.8 ± 5.6 | 98.8 ± 6.8 | 104.5 ± 7.4 | 97.9 ± 4.6 | 0.20 |
| SpO_2_ (%) | 96.5 ± 0.5 | 95.9 ± 1.3 | 94.8 ± 1.7 | 96.2 ± 1.7 | 0.07 |
| High intensity |  |  |  |  |  |
| Rf (breaths/min) | 32.2 ± 3.8 | 30.5 ± 3.8 | 30 ± 3.6 | 30.9 ± 6.0 | 0.74 |
| VE (L/min) | 51.7 ± 12.2 | 48.8 ± 16.2 | 47.4 ± 12.7 | 49.8 ± 13.1 | 0.94 |
| VCO_2_ (mL/min) | 1666.4±340.0 | 1655.8±470.9 | 1686.1±369.2 | 1669.5±406.1 | 1.00 |
| VO_2_/KG (mL/min/kg) | 26.3 ± 3.3 | 26.6 ± 5.8 | 27.5 ± 5.2 | 28.1 ± 2.3 | 0.90 |
| METs | 7.5 ± 0.9 | 7.6 ± 1.7 | 7.9 ± 1.5 | 8 ± 0.7 | 0.90 |
| HR (beats/min) | 125 ± 6.5 | 125.3 ± 8.1 | 129.9 ± 9.7 | 124.7 ± 1.4 | 0.53 |
| SpO_2_ (%) | 94.5 ± 2.5 | 93.8 ± 1.9 | 92.6 ± 3.5 | 93 ± 3.9 | 0.61 |
| Recovery |  |  |  |  |  |
| Rf (breaths/min) | 30.9 ± 2.7 | 28.9 ± 2.9 | 28 ± 2.0 | 29.1 ± 3.9 | 0.24 |
| VE (L/min) | 46.1 ± 11.8 | 44.5 ± 12.9 | 42.4 ± 10.5 | 43.6 ± 10.8 | 0.95 |
| VCO_2_ (mL/min) | 1407.9±287.2 | 1451.7±357.3 | 1462.5±295.3 | 1400.2±295.0 | 0.98 |
| VO_2_/KG (mL/min/kg) | 19 ± 2.2 | 19.9 ± 3.6 | 20.4 ± 2.8 | 19.4 ± 1.4 | 0.78 |
| METs | 5.4 ± 0.6 | 5.7 ± 1.0 | 5.8 ± 0.8 | 5.5 ± 0.4 | 0.78 |
| HR (beats/min) | 113.6 ± 8.9 | 118 ± 4.5 | 118.9 ± 7.3 | 111.8 ± 7.9 | 0.30 |
| SpO_2_ (%) | 96.4 ± 0.6 | 96.7 ± 1.4 | 96.4 ± 0.5 | 97.3 ± 1.2 | 0.55 |
| The values were shown in mean ± standard deviation. Significance level was set at p < 0.05. Significant results are indicated in bold.  Rf: Respiratory frequency; VE: Minute Ventilation; VCO_2_: Volume of Carbon dioxide consumed by the body per minute; VO_2_: Volume of Oxygen consumed by the body per minute; METs: Metabolic equivalent; HR: Heart Rate; SpO_2_: percutaneous oxygen saturation. | | | | | |
