# Supplementary material for: Physiological effects of filtering facepiece respirators based on age and exercise intensity
Source: PLoS One. 2024 Aug 29;19(8):e0309403. doi: 10.1371/journal.pone.0309403 (PMC11361601; doi:10.1371/journal.pone.0309403)
Supplement: S5 Table — (DOCX) [file pone.0309403.s005.docx]

| **S5 Table. Cohen’s and CI at various exercies intensities in children group.** | | | | |
| --- | --- | --- | --- | --- |
| Children group |  |  |  |  |
|  |  | | Cohen’s | CI 95% |
| Rest |  |  |  |  |
| Rf (breaths/min) | Control vs Cup | | -0.02 | (-0.92, 0.88) |
|  | Control vs FF | | 0.07 | (-0.84, 0.97) |
|  | Cup vs FF | | 0.09 | (-0.78, 0.97) |
| VE (L/min) | Control vs Cup | | -0.22 | (-1.12, 0.69) |
|  | Control vs FF | | 0.31 | (-0.6, 1.21) |
|  | Cup vs FF | | 0.52 | (-0.38, 1.4) |
| VCO_2_ (mL/min) | Control vs Cup | | -0.03 | (-0.93, 0.87) |
|  | Control vs FF | | 0.18 | (-0.73, 1.08) |
|  | Cup vs FF | | 0.20 | (-0.68, 1.08) |
| VO_2_/KG (mL/min/kg) | Control vs Cup | | -0.30 | (-1.2, 0.61) |
|  | Control vs FF | | -0.21 | (-1.11, 0.69) |
|  | Cup vs FF | | 0.07 | (-0.81, 0.94) |
| METs | Control vs Cup | | -0.30 | (-1.2, 0.61) |
|  | Control vs FF | | -0.22 | (-1.12, 0.69) |
|  | Cup vs FF | | 0.06 | (-0.82, 0.93) |
| HR (beats/min) | Control vs Cup | | -0.27 | (-1.17, 0.64) |
|  | Control vs FF | | -0.05 | (-0.95, 0.85) |
|  | Cup vs FF | | 0.18 | (-0.7, 1.06) |
| SpO_2_ (%) | Control vs Cup | | -1.16 | (-2.1, -0.19) |
|  | Control vs FF | | -0.46 | (-1.36, 0.46) |
|  | Cup vs FF | | 0.36 | (-0.55, 1.27) |
| Low intensity |  | |  |  |
| Rf (breaths/min) | Control vs Cup | | 0.11 | (-0.77, 0.98) |
|  | Control vs FF | | -0.04 | (-0.92, 0.83) |
|  | Cup vs FF | | -0.16 | (-1.03, 0.72) |
| VE (L/min) | Control vs Cup | | -0.38 | (-1.26, 0.51) |
|  | Control vs FF | | -0.26 | (-1.14, 0.62) |
|  | Cup vs FF | | 0.09 | (-0.79, 0.96) |
| VCO_2_ (mL/min) | Control vs Cup | | -0.22 | (-1.09, 0.67) |
|  | Control vs FF | | -0.40 | (-1.28, 0.49) |
|  | Cup vs FF | | -0.12 | (-1, 0.76) |
| VO_2_/KG (mL/min/kg) | Control vs Cup | | -0.11 | (-0.99, 0.76) |
|  | Control vs FF | | -0.49 | (-1.38, 0.4) |
|  | Cup vs FF | | -0.44 | (-1.32, 0.45) |
| METs | Control vs Cup | | -0.12 | (-0.99, 0.76) |
|  | Control vs FF | | -0.50 | (-1.38, 0.4) |
|  | Cup vs FF | | -0.44 | (-1.33, 0.45) |
| HR (beats/min) | Control vs Cup | | 0.11 | (-0.77, 0.99) |
|  | Control vs FF | | -0.19 | (-1.06, 0.69) |
|  | Cup vs FF | | -0.26 | (-1.14, 0.62) |
| SpO_2_ (%) | Control vs Cup | | 0.00 | (-0.88, 0.88) |
|  | Control vs FF | | 0.46 | (-0.43, 1.35) |
|  | Cup vs FF | | 0.51 | (-0.38, 1.4) |
| Moderate intensity |  | |  |  |
| Rf (breaths/min) | Control vs Cup | | 0.47 | (-0.43, 1.35) |
|  | Control vs FF | | -0.03 | (-0.91, 0.84) |
|  | Cup vs FF | | -0.52 | (-1.4, 0.38) |
| VE (L/min) | Control vs Cup | | -0.14 | (-1.02, 0.74) |
|  | Control vs FF | | -0.18 | (-1.05, 0.7) |
|  | Cup vs FF | | -0.06 | (-0.93, 0.82) |
| VCO_2_ (mL/min) | Control vs Cup | | -0.18 | (-1.05, 0.7) |
|  | Control vs FF | | -0.42 | (-1.3, 0.47) |
|  | Cup vs FF | | -0.21 | (-1.09, 0.67) |
| VO_2_/KG (mL/min/kg) | Control vs Cup | | -0.19 | (-1.07, 0.69) |
|  | Control vs FF | | -0.58 | (-1.47, 0.33) |
|  | Cup vs FF | | -0.41 | (-1.29, 0.48) |
| METs | Control vs Cup | | -0.19 | (-1.06, 0.69) |
|  | Control vs FF | | -0.58 | (-1.47, 0.33) |
|  | Cup vs FF | | -0.42 | (-1.3, 0.48) |
| HR (beats/min) | Control vs Cup | | 0.11 | (-0.77, 0.98) |
|  | Control vs FF | | -0.35 | (-1.23, 0.54) |
|  | Cup vs FF | | -0.39 | (-1.27, 0.51) |
| SpO_2_ (%) | Control vs Cup | | 0.54 | (-0.39, 1.45) |
|  | Control vs FF | | 0.80 | (-0.13, 1.7) |
|  | Cup vs FF | | 0.28 | (-0.63, 1.18) |
| High intensity |  | |  |  |
| Rf (breaths/min) | Control vs Cup | | 0.76 | (-0.16, 1.66) |
|  | Control vs FF | | 0.13 | (-0.75, 1) |
|  | Cup vs FF | | -0.51 | (-1.39, 0.39) |
| VE (L/min) | Control vs Cup | | 0.37 | (-0.52, 1.25) |
|  | Control vs FF | | 0.01 | (-0.87, 0.88) |
|  | Cup vs FF | | -0.28 | (-1.16, 0.6) |
| VCO_2_ (mL/min) | Control vs Cup | | 0.06 | (-0.82, 0.93) |
|  | Control vs FF | | -0.37 | (-1.25, 0.52) |
|  | Cup vs FF | | -0.38 | (-1.26, 0.51) |
| VO_2_/KG (mL/min/kg) | Control vs Cup | | -0.02 | (-0.89, 0.86) |
|  | Control vs FF | | -0.51 | (-1.39, 0.39) |
|  | Cup vs FF | | -0.57 | (-1.46, 0.33) |
| METs | Control vs Cup | | -0.02 | (-0.89, 0.86) |
|  | Control vs FF | | -0.51 | (-1.39, 0.39) |
|  | Cup vs FF | | -0.57 | (-1.46, 0.33) |
| HR (beats/min) | Control vs Cup | | 0.55 | (-0.35, 1.44) |
|  | Control vs FF | | -0.05 | (-0.93, 0.83) |
|  | Cup vs FF | | -0.60 | (-1.49, 0.31) |
| SpO_2_ (%) | Control vs Cup | | -0.12 | (-1.11, 0.87) |
|  | Control vs FF | | 0.37 | (-0.57, 1.3) |
|  | Cup vs FF | | 0.49 | (-0.52, 1.49) |
| Recovery |  | |  |  |
| Rf (breaths/min) | Control vs Cup | | 0.71 | (-0.21, 1.6) |
|  | Control vs FF | | 0.39 | (-0.5, 1.27) |
|  | Cup vs FF | | -0.34 | (-1.22, 0.55) |
| VE (L/min) | Control vs Cup | | 0.31 | (-0.57, 1.19) |
|  | Control vs FF | | 0.20 | (-0.68, 1.08) |
|  | Cup vs FF | | -0.06 | (-0.94, 0.81) |
| VCO_2_ (mL/min) | Control vs Cup | | -0.01 | (-0.88, 0.87) |
|  | Control vs FF | | -0.16 | (-1.04, 0.72) |
|  | Cup vs FF | | -0.13 | (-1.01, 0.75) |
| VO_2_/KG (mL/min/kg) | Control vs Cup | | -0.20 | (-1.07, 0.68) |
|  | Control vs FF | | -0.35 | (-1.23, 0.54) |
|  | Cup vs FF | | -0.10 | (-0.98, 0.77) |
| METs | Control vs Cup | | -0.19 | (-1.07, 0.69) |
|  | Control vs FF | | -0.35 | (-1.23, 0.54) |
|  | Cup vs FF | | -0.10 | (-0.98, 0.77) |
| HR (beats/min) | Control vs Cup | | 0.59 | (-0.32, 1.47) |
|  | Control vs FF | | 0.13 | (-0.75, 1) |
|  | Cup vs FF | | -0.46 | (-1.34, 0.44) |
| SpO_2_ (%) | Control vs Cup | | 0.03 | (-0.87, 0.93) |
|  | Control vs FF | | 0.18 | (-0.71, 1.05) |
|  | Cup vs FF | | 0.18 | (-0.73, 1.08) |
| The 95% confidence interval (CI) represents the difference in means as listed in S2 Table. | | | | |
