# Supplementary material for: Physiological effects of filtering facepiece respirators based on age and exercise intensity
Source: PLoS One. 2024 Aug 29;19(8):e0309403. doi: 10.1371/journal.pone.0309403 (PMC11361601; doi:10.1371/journal.pone.0309403)
Supplement: S6 Table. Cohen’s and CI at various exercies intensities in young adults group — (DOCX) [file pone.0309403.s006.docx]

| **S6 Table. Cohen’s and CI at various exercies intensities in young adults group.** | | | | |
| --- | --- | --- | --- | --- |
| Young adults group |  |  |  |  |
|  |  | | Cohen’s | CI 95% |
| Rest |  |  |  |  |
| Rf (breaths/min) | Control vs Cup | | 0.05 | (-0.85, 0.95) |
|  | Control vs FF | | 0.36 | (-0.52, 1.24) |
|  | Cup vs FF | | 0.33 | (-0.58, 1.23) |
|  | Cup vs Valve | | 0.44 | (-0.62, 1.47) |
|  | FF vs Valve | | 0.08 | (-0.93, 1.09) |
| VE (L/min) | Control vs Cup | | -0.41 | (-1.32, 0.5) |
|  | Control vs FF | | -0.13 | (-1.01, 0.75) |
|  | Cup vs FF | | 0.29 | (-0.62, 1.2) |
|  | Cup vs Valve | | 0.17 | (-0.87, 1.2) |
|  | FF vs Valve | | -0.17 | (-1.18, 0.85) |
| VCO_2_ (mL/min) | Control vs Cup | | -0.04 | (-0.94, 0.86) |
|  | Control vs FF | | -0.45 | (-1.33, 0.44) |
|  | Cup vs FF | | -0.33 | (-1.23, 0.58) |
|  | Cup vs Valve | | 0.09 | (-0.94, 1.12) |
|  | FF vs Valve | | 0.52 | (-0.52, 1.54) |
| VO_2_/KG (mL/min/kg) | Control vs Cup | | 0.00 | (-0.91, 0.9) |
|  | Control vs FF | | -0.72 | (-1.61, 0.2) |
|  | Cup vs FF | | -0.79 | (-1.72, 0.15) |
|  | Cup vs Valve | | 0.20 | (-0.83, 1.24) |
|  | FF vs Valve | | 0.95 | (-0.14, 2.01) |
| METs | Control vs Cup | | 0.00 | (-0.9, 0.9) |
|  | Control vs FF | | -0.70 | (-1.6, 0.21) |
|  | Cup vs FF | | -0.79 | (-1.71, 0.16) |
|  | Cup vs Valve | | 0.19 | (-0.84, 1.23) |
|  | FF vs Valve | | 0.93 | (-0.15, 1.99) |
| HR (beats/min) | Control vs Cup | | -0.36 | (-1.26, 0.56) |
|  | Control vs FF | | -0.19 | (-1.07, 0.69) |
|  | Cup vs FF | | 0.15 | (-0.75, 1.05) |
|  | Cup vs Valve | | -0.09 | (-1.12, 0.95) |
|  | FF vs Valve | | -0.26 | (-1.28, 0.76) |
| SpO_2_ (%) | Control vs Cup | | -0.83 | (-1.76, 0.12) |
|  | Control vs FF | | 0.10 | (-0.8, 1) |
|  | Cup vs FF | | 1.01 | (0.01, 1.99) |
|  | Cup vs Valve | | 0.71 | (-0.37, 1.77) |
|  | FF vs Valve | | -0.41 | (-1.44, 0.65) |
| Low intensity |  | |  |  |
| Rf (breaths/min) | Control vs Cup | | 0.73 | (-0.19, 1.63) |
|  | Control vs FF | | 0.51 | (-0.39, 1.4) |
|  | Cup vs FF | | -0.28 | (-1.16, 0.61) |
|  | Cup vs Valve | | -0.09 | (-1.1, 0.93) |
|  | FF vs Valve | | 0.19 | (-0.83, 1.2) |
| VE (L/min) | Control vs Cup | | 0.03 | (-0.85, 0.9) |
|  | Control vs FF | | 0.10 | (-0.78, 0.98) |
|  | Cup vs FF | | 0.07 | (-0.81, 0.95) |
|  | Cup vs Valve | | 0.13 | (-0.88, 1.14) |
|  | FF vs Valve | | 0.06 | (-0.95, 1.07) |
| VCO_2_ (mL/min) | Control vs Cup | | 0.00 | (-0.88, 0.87) |
|  | Control vs FF | | -0.37 | (-1.24, 0.52) |
|  | Cup vs FF | | -0.35 | (-1.23, 0.53) |
|  | Cup vs Valve | | 0.19 | (-0.83, 1.2) |
|  | FF vs Valve | | 0.59 | (-0.46, 1.61) |
| VO_2_/KG (mL/min/kg) | Control vs Cup | | 0.21 | (-0.67, 1.09) |
|  | Control vs FF | | -0.71 | (-1.61, 0.2) |
|  | Cup vs FF | | -0.88 | (-1.79, 0.05) |
|  | Cup vs Valve | | 0.39 | (-0.64, 1.4) |
|  | FF vs Valve | | 1.16 | (0.04, 2.24) |
| METs | Control vs Cup | | 0.22 | (-0.66, 1.09) |
|  | Control vs FF | | -0.71 | (-1.61, 0.2) |
|  | Cup vs FF | | -0.89 | (-1.8, 0.05) |
|  | Cup vs Valve | | 0.38 | (-0.65, 1.39) |
|  | FF vs Valve | | 1.15 | (0.04, 2.23) |
| HR (beats/min) | Control vs Cup | | -0.44 | (-1.32, 0.46) |
|  | Control vs FF | | 0.05 | (-0.85, 0.95) |
|  | Cup vs FF | | 0.46 | (-0.46, 1.37) |
|  | Cup vs Valve | | -0.01 | (-1.02, 1) |
|  | FF vs Valve | | -0.61 | (-1.66, 0.46) |
| SpO_2_ (%) | Control vs Cup | | 0.57 | (-0.34, 1.45) |
|  | Control vs FF | | 0.49 | (-0.41, 1.37) |
|  | Cup vs FF | | -0.06 | (-0.94, 0.82) |
|  | Cup vs Valve | | -0.62 | (-1.65, 0.42) |
|  | FF vs Valve | | -0.54 | (-1.56, 0.5) |
| Moderate intensity |  | |  |  |
| Rf (breaths/min) | Control vs Cup | | 0.54 | (-0.36, 1.43) |
|  | Control vs FF | | 0.45 | (-0.45, 1.33) |
|  | Cup vs FF | | -0.13 | (-1.01, 0.75) |
|  | Cup vs Valve | | -0.27 | (-1.29, 0.75) |
|  | FF vs Valve | | -0.16 | (-1.17, 0.86) |
| VE (L/min) | Control vs Cup | | 0.21 | (-0.67, 1.09) |
|  | Control vs FF | | 0.25 | (-0.63, 1.13) |
|  | Cup vs FF | | 0.05 | (-0.83, 0.93) |
|  | Cup vs Valve | | 0.09 | (-0.92, 1.11) |
|  | FF vs Valve | | 0.04 | (-0.98, 1.05) |
| VCO_2_ (mL/min) | Control vs Cup | | 0.05 | (-0.82, 0.93) |
|  | Control vs FF | | -0.25 | (-1.12, 0.64) |
|  | Cup vs FF | | -0.31 | (-1.18, 0.58) |
|  | Cup vs Valve | | 0.15 | (-0.86, 1.17) |
|  | FF vs Valve | | 0.46 | (-0.58, 1.48) |
| VO_2_/KG (mL/min/kg) | Control vs Cup | | 0.17 | (-0.71, 1.04) |
|  | Control vs FF | | -0.32 | (-1.2, 0.57) |
|  | Cup vs FF | | -0.45 | (-1.33, 0.44) |
|  | Cup vs Valve | | 0.25 | (-0.77, 1.26) |
|  | FF vs Valve | | 0.70 | (-0.36, 1.73) |
| METs | Control vs Cup | | 0.17 | (-0.71, 1.04) |
|  | Control vs FF | | -0.32 | (-1.2, 0.57) |
|  | Cup vs FF | | -0.45 | (-1.33, 0.44) |
|  | Cup vs Valve | | 0.24 | (-0.78, 1.26) |
|  | FF vs Valve | | 0.70 | (-0.36, 1.73) |
| HR (beats/min) | Control vs Cup | | -0.35 | (-1.23, 0.54) |
|  | Control vs FF | | 0.07 | (-0.83, 0.97) |
|  | Cup vs FF | | 0.34 | (-0.57, 1.25) |
|  | Cup vs Valve | | -0.12 | (-1.13, 0.9) |
|  | FF vs Valve | | -0.40 | (-1.44, 0.65) |
| SpO_2_ (%) | Control vs Cup | | -0.24 | (-1.14, 0.67) |
|  | Control vs FF | | -0.31 | (-1.18, 0.58) |
|  | Cup vs FF | | -0.08 | (-0.98, 0.83) |
|  | Cup vs Valve | | 0.17 | (-0.86, 1.21) |
|  | FF vs Valve | | 0.24 | (-0.78, 1.25) |
| High intensity |  | |  |  |
| Rf (breaths/min) | Control vs Cup | | 0.12 | (-0.76, 1) |
|  | Control vs FF | | 0.35 | (-0.54, 1.22) |
|  | Cup vs FF | | 0.21 | (-0.67, 1.09) |
|  | Cup vs Valve | | 0.23 | (-0.79, 1.24) |
|  | FF vs Valve | | 0.03 | (-0.99, 1.04) |
| VE (L/min) | Control vs Cup | | 0.15 | (-0.73, 1.02) |
|  | Control vs FF | | 0.19 | (-0.69, 1.07) |
|  | Cup vs FF | | 0.05 | (-0.83, 0.93) |
|  | Cup vs Valve | | 0.32 | (-0.71, 1.33) |
|  | FF vs Valve | | 0.24 | (-0.78, 1.25) |
| VCO_2_ (mL/min) | Control vs Cup | | 0.03 | (-0.85, 0.91) |
|  | Control vs FF | | -0.30 | (-1.18, 0.59) |
|  | Cup vs FF | | -0.33 | (-1.21, 0.55) |
|  | Cup vs Valve | | 0.22 | (-0.8, 1.23) |
|  | FF vs Valve | | 0.55 | (-0.49, 1.57) |
| VO_2_/KG (mL/min/kg) | Control vs Cup | | 0.16 | (-0.72, 1.03) |
|  | Control vs FF | | -0.44 | (-1.32, 0.46) |
|  | Cup vs FF | | -0.59 | (-1.48, 0.32) |
|  | Cup vs Valve | | 0.23 | (-0.79, 1.24) |
|  | FF vs Valve | | 0.77 | (-0.29, 1.81) |
| METs | Control vs Cup | | 0.16 | (-0.72, 1.03) |
|  | Control vs FF | | -0.44 | (-1.32, 0.46) |
|  | Cup vs FF | | -0.59 | (-1.48, 0.32) |
|  | Cup vs Valve | | 0.23 | (-0.79, 1.24) |
|  | FF vs Valve | | 0.77 | (-0.29, 1.81) |
| HR (beats/min) | Control vs Cup | | -0.27 | (-1.14, 0.62) |
|  | Control vs FF | | 0.05 | (-0.85, 0.95) |
|  | Cup vs FF | | 0.29 | (-0.62, 1.19) |
|  | Cup vs Valve | | -0.10 | (-1.12, 0.91) |
|  | FF vs Valve | | -0.36 | (-1.39, 0.69) |
| SpO_2_ (%) | Control vs Cup | | 0.80 | (-0.17, 1.75) |
|  | Control vs FF | | 0.30 | (-0.61, 1.2) |
|  | Cup vs FF | | -0.67 | (-1.58, 0.27) |
|  | Cup vs Valve | | -0.53 | (-1.57, 0.53) |
|  | FF vs Valve | | 0.00 | (-1.01, 1.01) |
| Recovery |  | |  |  |
| Rf (breaths/min) | Control vs Cup | | 0.15 | (-0.73, 1.03) |
|  | Control vs FF | | 0.41 | (-0.48, 1.29) |
|  | Cup vs FF | | 0.21 | (-0.68, 1.08) |
|  | Cup vs Valve | | 0.51 | (-0.53, 1.53) |
|  | FF vs Valve | | 0.32 | (-0.7, 1.34) |
| VE (L/min) | Control vs Cup | | 0.34 | (-0.55, 1.22) |
|  | Control vs FF | | 0.31 | (-0.58, 1.18) |
|  | Cup vs FF | | -0.01 | (-0.88, 0.87) |
|  | Cup vs Valve | | 0.50 | (-0.54, 1.52) |
|  | FF vs Valve | | 0.43 | (-0.61, 1.44) |
| VCO_2_ (mL/min) | Control vs Cup | | 0.05 | (-0.83, 0.92) |
|  | Control vs FF | | -0.44 | (-1.33, 0.45) |
|  | Cup vs FF | | -0.52 | (-1.41, 0.38) |
|  | Cup vs Valve | | 0.40 | (-0.63, 1.42) |
|  | FF vs Valve | | 0.94 | (-0.15, 1.99) |
| VO_2_/KG (mL/min/kg) | Control vs Cup | | 0.06 | (-0.81, 0.94) |
|  | Control vs FF | | -1.04 | (-1.96, -0.09) |
|  | Cup vs FF | | -0.98 | (-1.9, -0.03) |
|  | Cup vs Valve | | 0.48 | (-0.55, 1.5) |
|  | FF vs Valve | | 1.49 | (0.32, 2.62) |
| METs | Control vs Cup | | 0.07 | (-0.81, 0.94) |
|  | Control vs FF | | -1.03 | (-1.96, -0.08) |
|  | Cup vs FF | | -0.97 | (-1.89, -0.03) |
|  | Cup vs Valve | | 0.48 | (-0.55, 1.5) |
|  | FF vs Valve | | 1.49 | (0.32, 2.62) |
| HR (beats/min) | Control vs Cup | | -0.37 | (-1.25, 0.52) |
|  | Control vs FF | | -0.06 | (-0.96, 0.84) |
|  | Cup vs FF | | 0.27 | (-0.64, 1.17) |
|  | Cup vs Valve | | 0.13 | (-0.88, 1.14) |
|  | FF vs Valve | | -0.17 | (-1.21, 0.86) |
| SpO_2_ (%) | Control vs Cup | | 0.26 | (-0.65, 1.16) |
|  | Control vs FF | | -0.14 | (-1.06, 0.79) |
|  | Cup vs FF | | -0.42 | (-1.33, 0.5) |
|  | Cup vs Valve | | -0.27 | (-1.28, 0.75) |
|  | FF vs Valve | | 0.16 | (-0.88, 1.19) |
| The 95% confidence interval (CI) represents the difference in means as listed in S3 Table. | | | | |
