# Supplementary material for: Physiological effects of filtering facepiece respirators based on age and exercise intensity
Source: PLoS One. 2024 Aug 29;19(8):e0309403. doi: 10.1371/journal.pone.0309403 (PMC11361601; doi:10.1371/journal.pone.0309403)
Supplement: S7 Table — (DOCX) [file pone.0309403.s007.docx]

| **S7 Table. Cohen’s and CI at various exercies intensities in older adults group.** | | | | |
| --- | --- | --- | --- | --- |
| Older adults group |  |  |  |  |
|  |  | | Cohen’s | CI 95% |
| Rest |  |  |  |  |
| Rf (breaths/min) | Control vs Cup | | -0.07 | (-1.05, 0.91) |
|  | Control vs FF | | 0.36 | (-0.63, 1.35) |
|  | Cup vs FF | | 0.49 | (-0.51, 1.48) |
|  | Cup vs Valve | | -0.02 | (-1.22, 1.18) |
|  | FF vs Valve | | -0.46 | (-1.66, 0.77) |
| VE (L/min) | Control vs Cup | | -0.64 | (-1.64, 0.38) |
|  | Control vs FF | | -0.08 | (-1.06, 0.9) |
|  | Cup vs FF | | 0.57 | (-0.44, 1.56) |
|  | Cup vs Valve | | 0.21 | (-1, 1.41) |
|  | FF vs Valve | | -0.44 | (-1.64, 0.79) |
| VCO_2_ (mL/min) | Control vs Cup | | -0.15 | (-1.13, 0.84) |
|  | Control vs FF | | 0.03 | (-0.95, 1.01) |
|  | Cup vs FF | | 0.16 | (-0.82, 1.14) |
|  | Cup vs Valve | | 0.56 | (-0.68, 1.77) |
|  | FF vs Valve | | 0.45 | (-0.78, 1.65) |
| VO_2_/KG (mL/min/kg) | Control vs Cup | | -0.21 | (-1.19, 0.78) |
|  | Control vs FF | | -0.21 | (-1.19, 0.77) |
|  | Cup vs FF | | -0.04 | (-1.02, 0.94) |
|  | Cup vs Valve | | 0.39 | (-0.83, 1.6) |
|  | FF vs Valve | | 0.34 | (-0.87, 1.54) |
| METs | Control vs Cup | | -0.23 | (-1.21, 0.76) |
|  | Control vs FF | | -0.20 | (-1.18, 0.78) |
|  | Cup vs FF | | -0.01 | (-0.99, 0.97) |
|  | Cup vs Valve | | 0.40 | (-0.82, 1.61) |
|  | FF vs Valve | | 0.33 | (-0.89, 1.53) |
| HR (beats/min) | Control vs Cup | | -0.45 | (-1.44, 0.55) |
|  | Control vs FF | | -0.90 | (-1.93, 0.14) |
|  | Cup vs FF | | -0.32 | (-1.3, 0.67) |
|  | Cup vs Valve | | 0.51 | (-0.72, 1.72) |
|  | FF vs Valve | | 0.89 | (-0.39, 2.13) |
| SpO_2_ (%) | Control vs Cup | | 0.46 | (-0.54, 1.44) |
|  | Control vs FF | | 1.13 | (0.05, 2.18) |
|  | Cup vs FF | | 0.76 | (-0.27, 1.77) |
|  | Cup vs Valve | | -0.46 | (-1.67, 0.77) |
|  | FF vs Valve | | -1.10 | (-2.37, 0.21) |
| Low intensity |  | |  |  |
| Rf (breaths/min) | Control vs Cup | | 0.79 | (-0.24, 1.8) |
|  | Control vs FF | | 0.94 | (-0.11, 1.97) |
|  | Cup vs FF | | 0.20 | (-0.79, 1.18) |
|  | Cup vs Valve | | -0.27 | (-1.47, 0.94) |
|  | FF vs Valve | | -0.42 | (-1.63, 0.8) |
| VE (L/min) | Control vs Cup | | 0.00 | (-0.98, 0.98) |
|  | Control vs FF | | 0.26 | (-0.73, 1.24) |
|  | Cup vs FF | | 0.24 | (-0.75, 1.22) |
|  | Cup vs Valve | | 0.09 | (-1.11, 1.29) |
|  | FF vs Valve | | -0.16 | (-1.36, 1.04) |
| VCO_2_ (mL/min) | Control vs Cup | | 0.08 | (-0.9, 1.06) |
|  | Control vs FF | | 0.09 | (-0.9, 1.06) |
|  | Cup vs FF | | -0.01 | (-0.99, 0.97) |
|  | Cup vs Valve | | 0.18 | (-1.03, 1.37) |
|  | FF vs Valve | | 0.25 | (-0.96, 1.45) |
| VO_2_/KG (mL/min/kg) | Control vs Cup | | 0.28 | (-0.71, 1.26) |
|  | Control vs FF | | -0.05 | (-1.03, 0.93) |
|  | Cup vs FF | | -0.28 | (-1.26, 0.71) |
|  | Cup vs Valve | | -0.13 | (-1.33, 1.08) |
|  | FF vs Valve | | 0.20 | (-1.01, 1.4) |
| METs | Control vs Cup | | 0.28 | (-0.71, 1.26) |
|  | Control vs FF | | -0.04 | (-1.02, 0.94) |
|  | Cup vs FF | | -0.27 | (-1.25, 0.72) |
|  | Cup vs Valve | | -0.12 | (-1.32, 1.08) |
|  | FF vs Valve | | 0.20 | (-1.01, 1.4) |
| HR (beats/min) | Control vs Cup | | -0.36 | (-1.34, 0.63) |
|  | Control vs FF | | -0.82 | (-1.83, 0.22) |
|  | Cup vs FF | | -0.46 | (-1.45, 0.54) |
|  | Cup vs Valve | | 0.68 | (-0.57, 1.9) |
|  | FF vs Valve | | 1.07 | (-0.24, 2.34) |
| SpO_2_ (%) | Control vs Cup | | 0.73 | (-0.3, 1.73) |
|  | Control vs FF | | 0.95 | (-0.11, 1.97) |
|  | Cup vs FF | | 0.00 | (-0.98, 0.98) |
|  | Cup vs Valve | | -0.47 | (-1.67, 0.76) |
|  | FF vs Valve | | -0.57 | (-1.79, 0.67) |
| Moderate intensity |  | |  |  |
| Rf (breaths/min) | Control vs Cup | | 0.90 | (-0.15, 1.92) |
|  | Control vs FF | | 0.85 | (-0.19, 1.86) |
|  | Cup vs FF | | 0.02 | (-0.96, 1) |
|  | Cup vs Valve | | -0.49 | (-1.7, 0.74) |
|  | FF vs Valve | | -0.47 | (-1.68, 0.76) |
| VE (L/min) | Control vs Cup | | 0.25 | (-0.74, 1.23) |
|  | Control vs FF | | 0.35 | (-0.65, 1.33) |
|  | Cup vs FF | | 0.05 | (-0.93, 1.03) |
|  | Cup vs Valve | | -0.21 | (-1.41, 1) |
|  | FF vs Valve | | -0.30 | (-1.5, 0.92) |
| VCO_2_ (mL/min) | Control vs Cup | | 0.21 | (-0.78, 1.19) |
|  | Control vs FF | | 0.06 | (-0.92, 1.04) |
|  | Cup vs FF | | -0.15 | (-1.13, 0.83) |
|  | Cup vs Valve | | -0.17 | (-1.37, 1.04) |
|  | FF vs Valve | | -0.03 | (-1.23, 1.17) |
| VO_2_/KG (mL/min/kg) | Control vs Cup | | 0.30 | (-0.7, 1.28) |
|  | Control vs FF | | -0.09 | (-1.07, 0.89) |
|  | Cup vs FF | | -0.33 | (-1.31, 0.66) |
|  | Cup vs Valve | | -0.60 | (-1.82, 0.64) |
|  | FF vs Valve | | -0.24 | (-1.44, 0.97) |
| METs | Control vs Cup | | 0.30 | (-0.69, 1.28) |
|  | Control vs FF | | -0.10 | (-1.08, 0.88) |
|  | Cup vs FF | | -0.34 | (-1.32, 0.65) |
|  | Cup vs Valve | | -0.60 | (-1.82, 0.64) |
|  | FF vs Valve | | -0.23 | (-1.43, 0.98) |
| HR (beats/min) | Control vs Cup | | 0.00 | (-0.98, 0.98) |
|  | Control vs FF | | -0.88 | (-1.9, 0.16) |
|  | Cup vs FF | | -0.81 | (-1.82, 0.23) |
|  | Cup vs Valve | | 0.15 | (-1.06, 1.35) |
|  | FF vs Valve | | 1.00 | (-0.3, 2.26) |
| SpO_2_ (%) | Control vs Cup | | 0.65 | (-0.37, 1.65) |
|  | Control vs FF | | 1.41 | (0.28, 2.5) |
|  | Cup vs FF | | 0.76 | (-0.27, 1.77) |
|  | Cup vs Valve | | -0.27 | (-1.47, 0.94) |
|  | FF vs Valve | | -0.89 | (-2.13, 0.39) |
| High intensity |  | |  |  |
| Rf (breaths/min) | Control vs Cup | | 0.46 | (-0.55, 1.44) |
|  | Control vs FF | | 0.59 | (-0.42, 1.58) |
|  | Cup vs FF | | 0.12 | (-0.86, 1.1) |
|  | Cup vs Valve | | -0.10 | (-1.3, 1.1) |
|  | FF vs Valve | | -0.20 | (-1.4, 1.01) |
| VE (L/min) | Control vs Cup | | 0.20 | (-0.79, 1.18) |
|  | Control vs FF | | 0.34 | (-0.65, 1.33) |
|  | Cup vs FF | | 0.10 | (-0.89, 1.08) |
|  | Cup vs Valve | | -0.06 | (-1.26, 1.14) |
|  | FF vs Valve | | -0.18 | (-1.38, 1.03) |
| VCO_2_ (mL/min) | Control vs Cup | | 0.03 | (-0.95, 1.01) |
|  | Control vs FF | | -0.06 | (-1.03, 0.93) |
|  | Cup vs FF | | -0.07 | (-1.05, 0.91) |
|  | Cup vs Valve | | -0.03 | (-1.23, 1.17) |
|  | FF vs Valve | | 0.04 | (-1.16, 1.24) |
| VO_2_/KG (mL/min/kg) | Control vs Cup | | -0.06 | (-1.04, 0.92) |
|  | Control vs FF | | -0.28 | (-1.26, 0.71) |
|  | Cup vs FF | | -0.17 | (-1.14, 0.82) |
|  | Cup vs Valve | | -0.31 | (-1.51, 0.91) |
|  | FF vs Valve | | -0.14 | (-1.34, 1.07) |
| METs | Control vs Cup | | -0.06 | (-1.04, 0.92) |
|  | Control vs FF | | -0.27 | (-1.26, 0.72) |
|  | Cup vs FF | | -0.17 | (-1.15, 0.82) |
|  | Cup vs Valve | | -0.31 | (-1.51, 0.91) |
|  | FF vs Valve | | -0.14 | (-1.33, 1.07) |
| HR (beats/min) | Control vs Cup | | -0.03 | (-1.01, 0.95) |
|  | Control vs FF | | -0.59 | (-1.58, 0.43) |
|  | Cup vs FF | | -0.52 | (-1.5, 0.49) |
|  | Cup vs Valve | | 0.08 | (-1.12, 1.28) |
|  | FF vs Valve | | 0.64 | (-0.61, 1.85) |
| SpO_2_ (%) | Control vs Cup | | 0.34 | (-0.66, 1.32) |
|  | Control vs FF | | 0.61 | (-0.4, 1.61) |
|  | Cup vs FF | | 0.40 | (-0.6, 1.38) |
|  | Cup vs Valve | | 0.28 | (-0.93, 1.48) |
|  | FF vs Valve | | -0.10 | (-1.3, 1.1) |
| Recovery |  | |  |  |
| Rf (breaths/min) | Control vs Cup | | 0.73 | (-0.3, 1.74) |
|  | Control vs FF | | 1.22 | (0.08, 2.31) |
|  | Cup vs FF | | 0.36 | (-0.67, 1.38) |
|  | Cup vs Valve | | -0.06 | (-1.26, 1.14) |
|  | FF vs Valve | | -0.40 | (-1.63, 0.86) |
| VE (L/min) | Control vs Cup | | 0.13 | (-0.85, 1.11) |
|  | Control vs FF | | 0.32 | (-0.7, 1.34) |
|  | Cup vs FF | | 0.17 | (-0.85, 1.18) |
|  | Cup vs Valve | | 0.07 | (-1.13, 1.27) |
|  | FF vs Valve | | -0.11 | (-1.34, 1.12) |
| VCO_2_ (mL/min) | Control vs Cup | | -0.14 | (-1.11, 0.85) |
|  | Control vs FF | | -0.19 | (-1.2, 0.83) |
|  | Cup vs FF | | -0.03 | (-1.05, 0.98) |
|  | Cup vs Valve | | 0.15 | (-1.05, 1.35) |
|  | FF vs Valve | | 0.21 | (-1.03, 1.44) |
| VO_2_/KG (mL/min/kg) | Control vs Cup | | -0.28 | (-1.26, 0.71) |
|  | Control vs FF | | -0.58 | (-1.61, 0.47) |
|  | Cup vs FF | | -0.19 | (-1.2, 0.83) |
|  | Cup vs Valve | | 0.14 | (-1.06, 1.34) |
|  | FF vs Valve | | 0.43 | (-0.82, 1.67) |
| METs | Control vs Cup | | -0.29 | (-1.27, 0.71) |
|  | Control vs FF | | -0.58 | (-1.61, 0.47) |
|  | Cup vs FF | | -0.19 | (-1.2, 0.83) |
|  | Cup vs Valve | | 0.14 | (-1.06, 1.34) |
|  | FF vs Valve | | 0.43 | (-0.82, 1.66) |
| HR (beats/min) | Control vs Cup | | -0.61 | (-1.61, 0.4) |
|  | Control vs FF | | -0.64 | (-1.67, 0.42) |
|  | Cup vs FF | | -0.15 | (-1.17, 0.86) |
|  | Cup vs Valve | | 1.08 | (-0.23, 2.35) |
|  | FF vs Valve | | 0.95 | (-0.38, 2.23) |
| SpO_2_ (%) | Control vs Cup | | -0.28 | (-1.43, 0.88) |
|  | Control vs FF | | -0.05 | (-1.2, 1.1) |
|  | Cup vs FF | | 0.27 | (-0.79, 1.32) |
|  | Cup vs Valve | | -0.47 | (-1.82, 0.92) |
|  | FF vs Valve | | -1.22 | (-2.67, 0.28) |
| The 95% confidence interval (CI) represents the difference in means as listed in S4 Table. | | | | |
