# Supplementary material for: Physiological effects of filtering facepiece respirators based on age and exercise intensity
Source: PLoS One. 2024 Aug 29;19(8):e0309403. doi: 10.1371/journal.pone.0309403 (PMC11361601; doi:10.1371/journal.pone.0309403)
Supplement: S1 Fig — (a) Physiological indicators. (b) O2 and CO2 concentrations, based on the exercise intensity. (DOCX) [file pone.0309403.s008.docx]

| 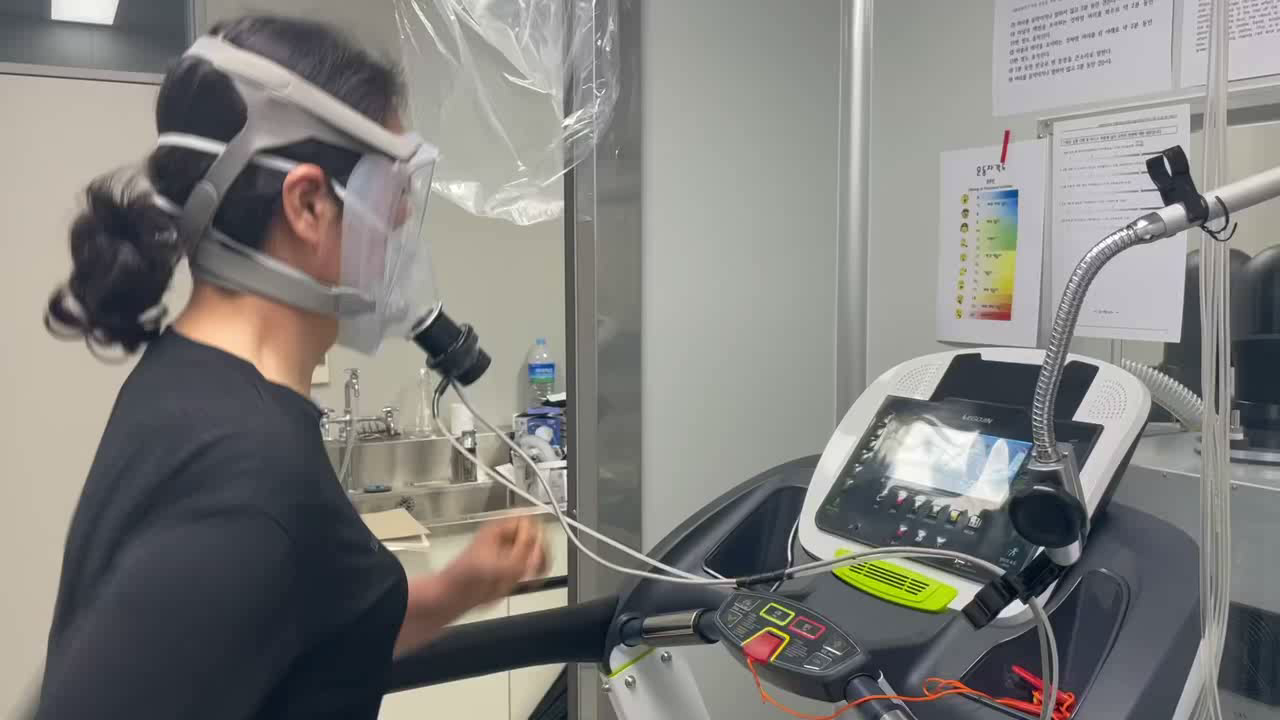  (a) |
| --- |
| 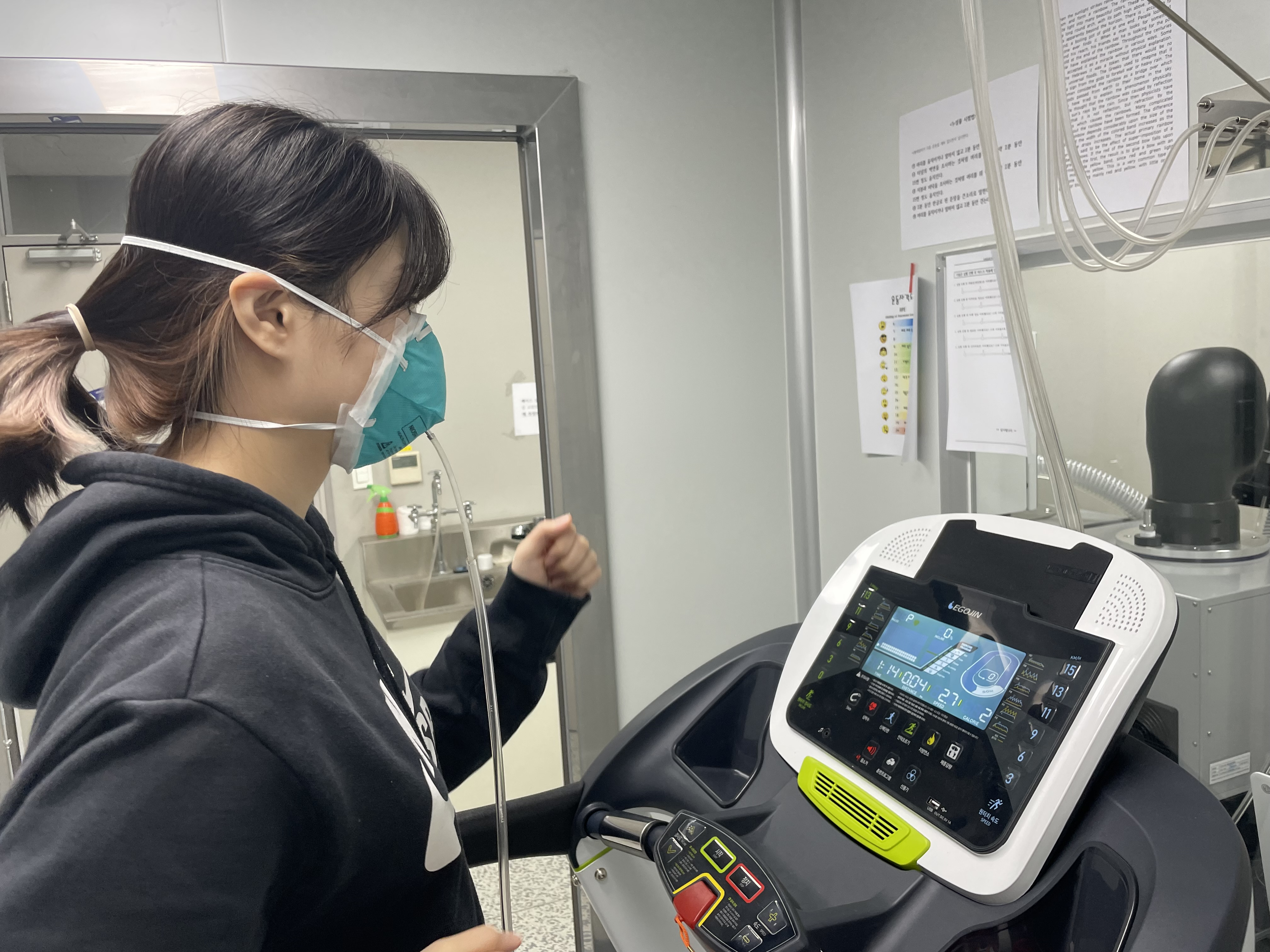  (b) |
| **S1 Fig. A photograph of a participant under assessment**. (a) Physiological indicators. (b) O_2_ and CO_2_ concentrations, based on the exercise intensity. |
