# Supplementary figures and images for: Physiological effects of filtering facepiece respirators based on age and exercise intensity
Source: PLoS One. 2024 Aug 29;19(8):e0309403. doi: 10.1371/journal.pone.0309403 (PMC11361601; doi:10.1371/journal.pone.0309403)

| 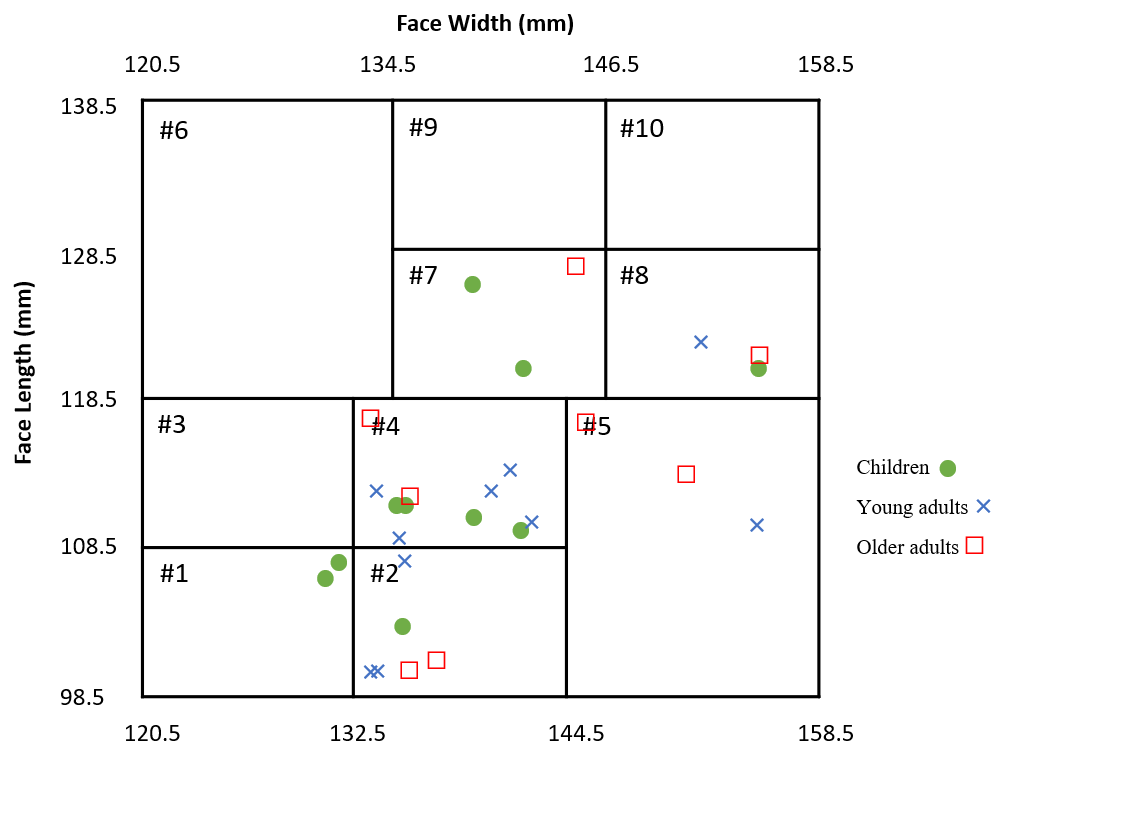 |
| --- |
| **S2 Fig. The distribution of participants in NIOSH panels.** |

Supplement: S2 Fig — (DOCX) [file pone.0309403.s009.docx]
