# Supplementary material for: Physiological effects of filtering facepiece respirators based on age and exercise intensity
Source: PLoS One. 2024 Aug 29;19(8):e0309403. doi: 10.1371/journal.pone.0309403 (PMC11361601; doi:10.1371/journal.pone.0309403)
Supplement: Attachment — Submitted filename: Response to reviewers_final.doc [file pone.0309403.s012.doc]

#### Reviewers’ comments and response

**▶** **No.: PONE-D-24-11410**

▶ **Title: Physiological Effects of Filtering Facepiece Respirators based on Age and Exercise Intensity**

**Response to editor’s comments**

All instructions from the editor regarding the submission of revised files were followed. The revised manuscript was submitted in two formats: 'revised manuscript with changes marked' and 'revised manuscript with no changes marked,' as per the guide. In response to the questions and comments from the reviewers, point-by-point responses and corrections have been incorporated into the text.

**Reviewer #1**

This manuscript explores the physiological effects of FFR on participants of different age groups engaging in daily life or short-time exercise under varying levels of exercise intensity using an improved mask. Its results support the conclusion that FFR has no substantial physiological impact on daily life or short-time exercise. However, before publication, the authors still need to address the following issues:
Main issues:

- ***Response***

Thank you for your insightful comments. Your comments highlighted points that we had not considered while writing the paper, and we have tried to answer each question sincerely, as shown below and in the revised manuscript. We are confident that your comments have greatly improved the quality of this paper. Please see the point-by-point responses to the comments.

Q1: There is insufficient participation from each age group, leading to low credibility of the analysis results. It is suggested that the authors continue to increase the number of participants.

- ***Response***

We have considered your comment regarding the number of participants seriously. As you suggested, increasing the number of participants will enhance the reliability of the data. However, for participatory pilot studies, especially those involving humans, it is challenging to select a large number of participants.

In this study, total 28 participants, with each age group consisted of 10 to 8 participants. Each participant wore a different mask and performed the experiment three times, making the actual content of the experiment quite extensive.

Below is a graph showing the number of participants in the 43 papers cited in the 43 participant studies analyzed in the meta-analysis of Reference 17 (reanalyzed Table 1 of the corresponding reference). As shown in the graph, this study had 28 participants, but there were 31 studies with fewer participants and 12 studies with more participants. Therefore, I do not believe that the number of participants in this study is very small.

Figure 1. Comparison of the number of participants in the 43 papers in the meta-analysis study in Reference 17 with the number of participants in this study.

If the number of participants becomes very large, the confidence interval narrows, which might affect the statistical significance. However, the difference in means will not be substantial.

Additionally, it is realistically challenging to continue this study further as the first author has a job.

Q2: Using a snowball sampling method may result in the obtained sample not representing the entire population well. The authors should describe the limitations of this recruitment method in the discussion.

- ***Response***

As the study was conducted in 2021 when the coronavirus was judged severe, the snowball sampling method was adopted. We have added a sentence in the discussion section to explain this as follows,

“Despite its advantages, the study has a few limitations. It was conducted in 2021 during a severe phase of the coronavirus pandemic, employing the snowball sampling method for recruitment and studying a relatively small number of subjects (n=28). Additionally, since all participants were healthy, the study may not fully represent the entire population, including patients with cardiovascular and neurological diseases. Caution should be exercised when interpreting and generalizing the data. Nevertheless, the study holds value for future larger-scale research focusing on the effects of FFRs during exercise across diverse age groups and varying exercise intensities.”

Q3: Have the authors excluded patients with neurological diseases such as myasthenia gravis or motor neuron diseases? These types of diseases may affect the results.

- ***Response***

Since it was an experiment in which subjects exercised at a high intensity while wearing an FFR, healthy subjects were selected to ensure the safety of the subjects. However, this study did not conduct medical screening for myasthenia gravis or motor neuron diseases. Since all participants reported that they were healthy and completed the given high-intensity exercise, we believe that they do not have these types of diseases.

Just like the answer in Question 2, I've added the following sentence to the discussion section to explain this as follows,

“Additionally, since all participants were healthy, the study may not fully represent the entire population, including patients with cardiovascular and neurological diseases. Caution should be exercised when interpreting and generalizing the data.”

Q4: The authors should provide the criteria for distinguishing between children, young adults, and older adults individuals.

- ***Response***

Section 3.1 provides demographic and anthropometric data, including age distribution across groups.

“All participant age groups had the same sex ratio; children were early adolescents, young adults were in their 20s and 30s, and older adults were in their 50s (Table 3).”

Also, according to your comment, we have revised the manuscript.

“Participants in the study were grouped based on age: children (five male and five female, aged 6–12 years), young adults (five male and five female, aged 24–34 years), and older adults (four male and four female, aged 51–60 years).”

However, this paper does not use the usual age classifications: children under 18 years of age, young adults from 18 to approximately 25-30 years, middle adulthood from around 30 to 50 years, and late adulthood from about 50 to 65 years or older. The reason is that a specific age range is concentrated within each group (e.g., children aged 11 to 12)

Miner issues:

Q1: The p-values in Table 4 should be presented in italics, and the formatting of the dashed lines used for grouping should be consistent.

- ***Response***

According to your comment, we have revised the manuscript.

Q2: The writing format throughout the manuscript (including figures and tables) should be standardized. For example, "young adults" and "older adults" should be consistently written in the same format.

- ***Response***

According to your comment, we have revised the manuscript.

**Reviewer #3**

I have had the opportunity to review your study titled " Physiological Effects of Filtering Facepiece Respirators based on Age and Exercise Intensity" aims to evaluate the physiological effects of different types of Filtering Facepiece Respirators (FFRs) across various age groups and exercise intensities. The study investigates parameters such as respiratory frequency, minute ventilation, carbon dioxide production, oxygen consumption, heart rate, metabolic equivalents, and percutaneous oxygen saturation. There is no specific fault in the given text. However, here are some comments and suggestions:

- ***Response***

Thank you for your insightful comments. Your comments highlighted points that we had not considered while writing the paper, and we have tried to answer each question sincerely, as shown below and in the revised manuscript. We are confident that your comments have greatly improved the quality of this paper. Please see the point-by-point responses to the comments.

Q1: There are some typographical errors like, In the abstract: "Filtering Facepiece 26 Respirators (FFRs)" should be "Filtering Facepiece Respirators (FFRs)." "FFRs was one of the most effective measures" should be "FFRs were one of the most effective measures."

- ***Response***

According to your comment, we have revised the manuscript.

Q2: Ensure consistent use of past tense throughout the abstract and manuscript.

- ***Response***

According to your comment, we have revised the manuscript.

Here's an example:

Before: The discontinuation criterion is met when the respiratory exchange ratio (CO_2_ production/O_2_ intake) was >1.1 or when the participants requested for discontinuation of exercise

After: The discontinuation criterion was met when the respiratory exchange ratio (CO_2_ production/O_2_ intake) was >1.1 or when the participants requested for discontinuation of exercise

Q3: The methods section should discuss the statistical power and how the sample size (28 participants) was determined.

- ***Response***

Thank you for your inquiry. The sample size of 28 participants was determined based on practical considerations rather than a formal power analysis or statistical hypothesis testing. Given the constraints at the time of recruitment and the scope of our study, we aimed to include as many participants as feasible within our resources. Although the sample size is limited, we have acknowledged this limitation and interpreted our findings cautiously, considering the potential impact on statistical power and generalizability.

Your question is related to another reader's question, so I answered it as follows.

As the study was conducted in 2021 when the coronavirus was judged severe, the snowball sampling method was adopted. We have added a sentence in the discussion section to explain this as follows,

“Despite its advantages, the study has a few limitations. It was conducted in 2021 during a severe phase of the coronavirus pandemic, employing the snowball sampling method for recruitment and studying a relatively small number of subjects (n=28). Additionally, since all participants were healthy, the study may not fully represent the entire population, including patients with cardiovascular and neurological diseases. Caution should be exercised when interpreting and generalizing the data. Nevertheless, the study holds value for future larger-scale research focusing on the effects of FFRs during exercise across diverse age groups and varying exercise intensities.”

In addition, with regard to statistical power, the discussion section rather than the methods was supplemented as follows.

“Statistical power was considered to determine whether the design of this study was appropriate. Specifically, three types of FFRs, three age groups, gender, and five levels of exercise were used as independent variables. The three representative dependent variables—respiratory frequency, VCO2, and SpO2—were selected from Table S2, with the effect size for each shown as averages and standard deviations in Tables S2 to S4. When considering the deviation (e.g., a mean difference in respiratory frequency of 5 with a standard deviation of 2.5) and a significance level set at 0.05, the estimated statistical power for the effects on respiratory frequency, VCO2, and SpO2 under the given conditions was approximately 1.0 for each dependent variable. The fact that the power was very high, close to 1.0, indicates that the effect sizes were significant, the sample size was adequate, and the analysis conditions were well adjusted. However, the research results revealed no significant differences due to wearing an FFR, except for those dependent on age group.”

Q4: Clearly mentioned, but ensure it is consistently referenced throughout the manuscript.

- ***Response***

It's not clear what exactly your comment means. However, based on your and other reviewers' opinions, we revised the entire manuscript again to maintain consistency.

Q5: In data presentation, Include more visual representations (e.g., graphs) in the results section to complement the text and tables.

- ***Response***

We have taken your comment seriously. Currently, there are three pictures and five tables. We considered converting the tables into figures; however, the existing tables present more detailed data, so we did not create new figures. Any missing information in the text can be found in the Supplementary Information (SI). There was also a concern about having too many pictures, leading the authors to ultimately decide to leave the layout as is. We hope you understand this decision.

Q6: Address the small sample size and potential biases in participant selection more thoroughly in the discussion section.

- ***Response***

Thank you for your advice. As the study was conducted in 2021 when the coronavirus was judged severe, the snowball sampling method was adopted.

We have added a sentence in the discussion section to explain this as follows,

“Despite its advantages, the study has a few limitations. It was conducted in 2021 during a severe phase of the coronavirus pandemic, employing the snowball sampling method for recruitment and studying a relatively small number of subjects (n=28). Additionally, since all participants were healthy, the study may not fully represent the entire population, including patients with cardiovascular and neurological diseases. Caution should be exercised when interpreting and generalizing the data. Nevertheless, the study holds value for future larger-scale research focusing on the effects of FFRs during exercise across diverse age groups and varying exercise intensities.”

Q7: Elaborate on the practical implications of the findings in the discussion section, especially in the context of public health recommendations during the pandemic.

- ***Response***

Thank you for your idea. According to your comment, we have added a sentence in the discussion section to explain this as follows,

“The World Health Organization strongly recommends FFR-wearing as a crucial measure in preventing the transmission of COVID-19, supported by numerous studies and data [16]. Research indicates that FFRs can be effectively worn across various age groups and in diverse exercise environments, including high-intensity activities.”

**Added Reference.**

16. WHO. WHO Diseases-COVID-19. Available online: Advice for the public: Coronavirus disease (accessed on 24 June 2024).

Q8: Ensure consistent use of terminology throughout the manuscript (e.g., FFR, mask, respirator).

- ***Response***

Following your advice, we have maintained consistency with the term FFR throughout the manuscript.

**Reviewer #4**

**Title**: The title is clear and reflects the content of the study, indicating the focus on physiological effects of FFRs based on age and exercise intensity.

Thank you for your insightful comments. Your comments highlighted points that we had not considered while writing the paper, and we have tried to answer each question sincerely, as shown below and in the revised manuscript. We are confident that your comments have greatly improved the quality of this paper. Please see the point-by-point responses to the comments.

Abstract:

- The abstract lacks a complete and clear opening sentence to set the context.
- The methodology is overly detailed for an abstract, yet it provides essential information.
- Results are presented clearly but lack emphasis on statistical significance.
- The conclusion is unclear and should better specify the practical implications of the findings.
- ***Response***

The entire abstract has been rewritten to reflect your various opinions on the abstract. Please refer to the before and after changes below. I believe your comments have made the abstract clearer.

Before:

During the coronavirus disease 2019 pandemic, Filtering Facepiece Respirators (FFRs) was one of the most effective measures. Concerns over their physiological effects increased as all age groups began to wear FFRs. This study aimed to evaluate the physiological effects of FFRs based on age and various exercise intensities.

Children (n = 10), young adults (n = 10), and older individuals (n = 8) accounted for a total of 28 participants. Physiological effects, including respiratory frequency (Rf), minute ventilation (VE), carbon dioxide production (VCO_2_), oxygen consumption (VO_2_), heart rate (HR), metabolic equivalents (METs), percutaneous oxygen saturation (SpO_2_), and the concentration of O_2_ and CO_2_ in the FFRs were measured via cardiopulmonary exercise testing on a treadmill. Without FFR (control) and three different FFR designs, cup-shaped, flat-folded, and an FFR with an exhalation valve were used in this study.

There was no significant difference in physiological effects between the control and FFR types, although Rf, VE, VCO_2_, VO_2_, METs, and HR increased with increasing exercise intensity. Depending on the exercise intensity, the O_2_ level in the FFR dead space decreased, and the CO_2_ level increased but this was independent of the dead space volume or FFR type.

We found that FFR did not have a substantial physiological effect on daily life or short-time exercise.

After:

During the coronavirus disease 2019 pandemic, Filtering Facepiece Respirators (FFRs) were highly effective, but concerns arose regarding their physiological effects across different age groups. This study evaluated these effects based on age and exercise intensity in 28 participants (children, young adults, and older individuals).

Physiological parameters such as respiratory frequency (Rf), minute ventilation (VE), carbon dioxide production (VCO_2_), oxygen consumption (VO_2_), heart rate (HR), metabolic equivalents (METs), percutaneous oxygen saturation (SpO_2_) and the concentration of O_2_ and CO_2_ in the FFRs were measured during treadmill tests with and without FFRs (cup-shaped, flat-folded, and with an exhalation valve). There was no significant difference in physiological effects between the control and FFR types, although Rf, VE, VCO_2_, VO_2_, METs, and HR increased with increasing exercise intensity. Depending on the exercise intensity, the O_2_ level in the FFR dead space decreased, and the CO_2_ level increased but this was independent of the dead space volume or FFR type. The study concluded that FFRs did not substantially impact daily life or short-term exercise, supporting their safe and effective use as a public health measure during pandemics and informing inclusive guidelines and policies.

Q1: The sample size is relatively small (10 children, 10 young adults, and 8 older adults). This limited sample may not be representative of the general population and could affect the generalizability of the findings. The study used a convenience sampling method (snowball sampling and postings on bulletin boards), which might introduce selection bias.

- ***Response***

Another reviewer also asked a similar question about this part. We have considered your comment and another reviewer comment regarding the number of participants seriously. As you suggested, increasing the number of participants will enhance the reliability of the data. However, for participatory pilot studies, especially those involving humans, it is challenging to select a large number of participants.

In this study, total 28 participants, with each age group consisted of 10 to 8 participants. Each participant wore a different mask and performed the experiment three times, making the actual content of the experiment quite extensive.

Below is a graph showing the number of participants in the 43 papers cited in the 43 participant studies analyzed in the meta-analysis of Reference 17 (reanalyzed Table 1 of the corresponding reference). As shown in the graph, this study had 28 participants, but there were 31 studies with fewer participants and 12 studies with more participants. Therefore, I do not believe that the number of participants in this study is very small.

Figure 1. Comparison of the number of participants in the 43 papers in the meta-analysis study in Reference 17 with the number of participants in this study.

If the number of participants becomes very large, the confidence interval narrows, which might affect the statistical significance. However, the difference in means will not be substantial.

Additionally, it is realistically challenging to continue this study further as the first author has a job.

Thank you for your advice. As the study was conducted in 2021 when the coronavirus was judged severe, the snowball sampling method was adopted.

We have added a sentence in the discussion section to explain this as follows,

“Despite its advantages, the study has a few limitations. It was conducted in 2021 during a severe phase of the coronavirus pandemic, employing the snowball sampling method for recruitment and studying a relatively small number of subjects (n=28). Additionally, since all participants were healthy, the study may not fully represent the entire population, including patients with cardiovascular and neurological diseases. Caution should be exercised when interpreting and generalizing the data. Nevertheless, the study holds value for future larger-scale research focusing on the effects of FFRs during exercise across diverse age groups and varying exercise intensities.”

Additionally, the following sentence was inserted as a limitation in the discussion.

“Despite its advantages, the study has a few limitations. It was conducted in 2021 during a severe phase of the coronavirus pandemic, employing the snowball sampling method for recruitment and studying a relatively small number of subjects (n=28).”

Q2: While the use of ANOVA and paired t-tests is appropriate, the paper lacks a detailed justification for the chosen statistical methods. The results section reports p-values but does not provide confidence intervals or effect sizes, which are crucial for understanding the practical significance of the findings.The Bonferroni correction is mentioned, but its application is not clearly explained in the context of multiple comparisons.

- ***Response***

Before;

We performed the analysis of variance (ANOVA) and a paired t-test to determine statistical differences between the control and FFR types at various exercise intensities and calculated the differences in physiological variables for each exercise intensity interval.

After :

Measurements within each group were normally distributed, so parametric statistics were applied. A paired t-test was used to assess differences between two groups, and analysis of variance (ANOVA) was used to examine differences among three or more groups, testing the differences in physiological variables for each exercise intensity interval.

We also included CI 95% and the effect size tables in the Supporting Information (S5-7 Tables). Mention of the Bonferroni correction was also added to the tables (Tables 4-5).

Q3: Although the study controls for some potential confounders (e.g., excluding smokers and those with certain health conditions), other variables such as participants' baseline fitness levels and previous experience with wearing masks are not accounted for. The study does not report on adherence to pre-test instructions (e.g., avoiding caffeine and excessive physical activity), which could influence the results.

- ***Response***

Thank you for your advice. Table 3 provides demographic and anthropometric data, including the average weekly physical activity time

We have added a sentence in the method section to explain this as follows,

“Their average weekly physical activity time was also collected to assess baseline fitness levels.”

Also, Participants involved in the study describes about pre-test instructions.

According to your comment, we have revised the manuscript.

“Participants wore comfortable clothing and shoes during the experiment, refrained from excessive physical activity for 48 hours before each trial, and avoided consuming caffeinated beverages and food for 3 hours before each trial.”

Also, in connection with your previous question, I added the following sentence to the discussion section.

“Despite its advantages, the study has a few limitations. It was conducted in 2021 during a severe phase of the coronavirus pandemic, employing the snowball sampling method for recruitment and studying a relatively small number of subjects (n=28). Additionally, since all participants were healthy, the study may not fully represent the entire population, including patients with cardiovascular and neurological diseases. Caution should be exercised when interpreting and generalizing the data. Nevertheless, the study holds value for future larger-scale research focusing on the effects of FFRs during exercise across diverse age groups and varying exercise intensities.”

Q4: The paper mentions using different types of FFRs but does not provide a rationale for selecting these specific types.

The description of the dead space measurement and gas concentration analysis is somewhat unclear. More details on how these measurements were conducted and their accuracy would be beneficial.

- ***Response***

I have inserted the following as an answer to your question about the rationale for selecting other FFRs.

“The reason for selecting FFRs of different shapes is due to the variation in dead space size depending on the FFR design. Additionally, including valve types aims to assess the activation of air circulation within the dead space. The brands selected here are certified masks that were dominant in sales in online stores at the time of the study.”

Dead space measurement and gas concentration analysis are already described in the text as follows. In addition, citations to related literature were provided so that further references could be found.

**Added Reference.**

14. Geiss, O. Effect of Wearing Face Masks on the Carbon Dioxide Concentration in the Breathing Zone. Aerosol Air Qual. 2021;21(2):1-7. DOI: [10.4209/aaqr.2020.07.0403](https://doi.org/10.4209/aaqr.2020.07.0403)

Q5: Enhanced Discussion: Offer a more detailed interpretation of the results, considering both the study's strengths and limitations, and relate the findings to the broader literature.

- ***Response***

Thank you for your idea. According to your comment, we have revised the manuscript. to address its strengths and limitations.

“The World Health Organization strongly recommends FFR-wearing as a crucial measure in preventing the transmission of COVID-19, supported by numerous studies and data [16]. Research indicates that FFRs can be effectively worn across various age groups and in diverse exercise environments, including high-intensity activities.”

“Despite its advantages, the study has a few limitations. It was conducted in 2021 during a severe phase of the coronavirus pandemic, employing the snowball sampling method for recruitment and studying a relatively small number of subjects (n=28). Additionally, since all participants were healthy, the study may not fully represent the entire population, including patients with cardiovascular and neurological diseases. Caution should be exercised when interpreting and generalizing the data. Nevertheless, the study holds value for future larger-scale research focusing on the effects of FFRs during exercise across diverse age groups and varying exercise intensities.”

Miner issues:

Q1: Some sections, particularly the methods and results, are dense and could benefit from clearer subheadings and more concise explanations.

- ***Response***

According to your comment, we aimed to clarify the meaning by subdividing the sections into "Method" and "Results" as subheadings. We have revised the manuscript to achieve more concise explanations by editing and omitting certain sentences.

Before: 2.2 Filtering Facepiece Respirators

After: 2.2.1 Selection of FFR shape, 2.2.2 Measurement of dead space according to types of FFR

Q2: Figures and tables should be referenced more explicitly in the text, and their captions should provide enough information to be understood independently.

- ***Response***

According to your comment, we have revised the manuscript.

Here's an example:

Before: Table 2. Measurements of the physiological index according to exercise intensity

After: Table 2. Physiological indices measured across exercise intensities, along with O_2_ and CO_2_ concentrations, perceptual measures, and measurement time points
